# Construction of a Novel Lignin-Based Quaternary Ammonium Material with Excellent Corrosion Resistant Behavior and Its Application for Corrosion Protection

**DOI:** 10.3390/ma12111776

**Published:** 2019-05-31

**Authors:** Chao Gao, Shoujuan Wang, Xinyu Dong, Keyin Liu, Xin Zhao, Fangong Kong

**Affiliations:** State Key Laboratory of Biobased Material and Green Papermaking, Key Laboratory of Pulp & Paper Science and Technology of Shandong Province/Ministry of Education, Shandong Academy of Sciences, Qilu University of Technology, Jinan 250353, China; 15864029637@163.com (C.G.); nancy5921@163.com (S.W.); d18954536225@163.com (X.D.); keyinliu@163.com (K.L.)

**Keywords:** kraft lignin, polymer, corrosion resistant, iron, acid inhibition

## Abstract

A novel lignin-based quaternary ammonium material (lignin-DMC) with excellent corrosion resistant behavior was synthesized by grafting DMC (methacrylatoethyl trimethyl ammonium chloride) onto kraft lignin. The structure and anti-corrosion performance of lignin-DMC was investigated using many methods, for instance the scanning electron microscope (SEM), atomic force microscopy (AFM), charge density analysis, molecular weight analysis, electrochemical methods. Fourier-transform infrared spectroscopy (FT-IR) confirmed the formation of the lignin-DMC. The experiment results indicated that maximum corrosion inhibition efficiency (87.65%) occurred at a concentration of 75 mg/L via weight loss measurement. Polarization curves indicated that lignin-DMC was a mixed-type inhibitor with an efficient anti-corrosion performance in an acid medium. Electrochemical impedance spectroscopy (EIS) results indicated that lignin-DMC could create a shielding effectiveness and achieve a protective effectiveness in the HCl solution. Moreover, lignin-DMC displayed a physical and chemical adsorption process between 20 KJ/mol and 40 KJ/mol, which followed the Langmuir adsorption isotherm model.

## 1. Introduction

During daily production, metallic materials usually react with the contacted medium (air, climate, salt spray, solution, etc.), causing the metal products to suffer from various degrees of corrosion, especially the carbon steel, it is a mild and commonly used material. In an aggressive acidic solution, carbon steel will be destroyed, which leads to significant economic losses [1,2]. Many techniques, such as the cathodic protection method and electroplates, coating processes, have been applied to protect metal bases from corrosion [3,4,5]. Among all of these techniques, the inhibitors is one of the most useful ways [6,7,8,9,10,11,12]. Corrosion inhibitors are a class of substances that can effectively inhibit metal corrosion and protect metal materials by adding another small amount of material. However, compared with other methods, the use of corrosion inhibitors has the following advantages: First, it does not substantially change the corrosive environment, secondly, it does not substantially increase equipment investment. Third, the corrosion inhibition effect is not affected by the shape of the equipment and the same formulation can sometimes, at the same time, prevent corrosion of various metals in different environments. [13,14,15]. In general, inhibitors contain N, O, S and other heteroatoms that can be easily adsorbed onto the surface of iron and that then compete with the corrosive ions to reduce the contact between the corrosive substances and active sites to attain the effect of corrosion inhibition [16,17,18,19,20,21,22,23,24]. Since the inhibitor is less costly and has strong applicability, it is generally used in petroleum exploration, chemical cleaning, storage and the transportation of metal products. With improvements in the awareness of environmental protection, new requirements have also been put forward for the development and application of corrosion inhibitors. Researching and developing biological inhibitors that do not pose a destructive effect on the environment is the development direction of the future [25,26]. The corrosion inhibitors from natural plants and animals have the advantages of nontoxicity, low pollution, and low cost [27,28,29]. Therefore, natural substance could be seen as a promising source of corrosion inhibitor [30,31,32]. In the last few years, several naturally occurring polymers have been used as anti-corrosion in a corrosive environment, including starch, chitosan, cellulose, etc. [30,33,34,35,36]. Due to the existence of functional groups in natural polymers, the iron surface can be protected from corrosion.

Lignin is a byproduct of the pulping process. It is a threat to the environment if not handled properly. Lignin seems to have several current and potential applications in many fields [37,38], however, the application as an anti-corrosion material was rarely reported. As corrosion inhibitors, the main groups of lignin that are responsible in inhibiting corrosion are the hydroxyl, methyl and carboxyl groups, which can adsorb onto iron or other metal surfaces to slow down the corrosion rate [39]. However, due to the insolubility of lignin, the anti-corrosion behavior of lignin is always low. Therefore, it needs to be modified by chemicals to improve its the water-solubility and to also make it meet application requirements. In one study, acrylamide (AM) was grafted onto lignin to research its anti-corrosion performance [35]. Its inhibition efficiency was found to be 77.10% at a 100 mg/L concentration. In other study, Hussin and Rahim produced a successful inhibitor by 2-naphthol and 1,8-dihydroxyanthraquinone grafted with kraft lignin and the resulting efficiency was 60.77% when the concentration was 100 mg/L [40]. However, little work has been conducted in studying the corrosion inhibition of metal by lignin that has been chemically modified with methacrylatoethyl trimethyl ammonium chloride (DMC).

In this paper, kraft lignin was copolymerized with a cationic monomer, DMC, to prepare the lignin-DMC copolymer as a corrosion inhibitor. The inhabitation performance of the lignin-DMC inhibitor was investigated in detail by the weight-loss method, electrochemical, SEM, AFM.

## 2. Materials and Experimental Method

### 2.1. Steel Specimen Preparation 

The chemical composition of the iron-based materials was C 3.05%, Al 1.45%, Mg 0.39%, Si 0.49%, and balance Fe. The specimens were first burnished using various grades of emery papers from #60 to #500 and then washed, degreased with acetone and dried at room temperature.

### 2.2. Surface Analysis 

The morphology of the iron-based materials specimen before and after adding corrosion inhibitor into the HCl solution were measured via SEM (FEI NOVA NANO SEM 450, FEI company, Hillsboro, OH, USA) and AFM (Multimode 8, Bruker, Santa Barbara, CA, USA). The iron-based materials of 5.0 cm × 2.0 cm × 1.0 mm were polished and immersed in 1.0 mol/L HCl solutions with and without 75 mg/L of the lignin-DMC inhibitor at 25 °C. Afterwards, the specimens were taken out of the solution and rinsed with deionized water. The washed specimens were dried at 105 °C for 12 h.

### 2.3. Weight Loss Method

The weight loss of iron-based materials were evaluated after 2 h of immersion in 100 mL of 1 mol/L HCl solutions with and without different concentrations of the lignin-DMC at room temperature. The samples were taken out, washed, dried and accurately reweighed. The inhibition efficiency was calculated according to Equation (1) [41]

(1) $$\mathrm{IE}\;(\%)\;=\;\frac{\;\left(W_{0}-W^{\prime }\right)\;}{W_{0}}\times 100$$

The difference between W_0_ and W′ represents the weight loss of the specimen at various concentrations of inhibitor. The corrosion rate (CR) can be calculated using Equation (2) [42]

(2) $$\mathrm{CR}\;(\mathrm{mg}/({\mathrm{cm}}^{2}\cdot h))\;=\;\frac{\Delta W}{\mathrm{At}}$$

where ΔW is the weight loss, W_0_–W, mg; A is the exposed area of the specimen, cm^2^; and t is the exposed time, h [43].

### 2.4. Electrochemical Method

For the electrochemical method, we started at a usual three-electrode cell. A platinum filament was used as a counter electrode (PE), a saturated calomel electrode (SCE) was the reference electrode (RE). The iron-based material was deemed as the working electrode (WE) with an uncovered area of 1.00 cm^2^. The electrochemical impedance spectroscopy (EIS) was investigated by using the IVIUM electrochemical workstation (IviumTechnologies BV Co., Eindhoven, Noord-Brabant, Netherlands), made in the Netherlands. The process used for the polarization method is similar to other articles. At first, the work electrode was submerged in an HCl solution for 1 h to achieve a stable state to determine the OCP (Open Circuit Potential) before measurement. The scanning electric potential was ± 250 mV (compared to open circuit potential) OCP (compared to SCE), and the scanning frequency rate was 30 mV/s. All electrochemical parameters were obtained by Tafel lines. The IE (Inhibition Efficiency) results were based on Equation (3).

(3) $$\mathrm{IE}\;(\%)\;=\;\frac{\left(i\;\mathrm{corr}-i’\mathrm{corr}\right)\;}{\mathrm{icorr}}\;\times \;100$$

where icorr and i’corr stand for the corrosion current densities absent and with an inhibitor, separately.

EIS was measured at OCP and a frequency range of 0.01–100 k Hz, the amplitude of the AC (Alternating Current) signal was 10 mv. The date of the impedance data were found in the Nyquist plots by calculating the difference in the values of the impedance at different frequencies [44]. Then, the inhibition efficiency can be calculated by formula (4).

(4) $$\mathrm{IE}\;(\%)\;=\;\frac{\left(\mathrm{Rct}\;–R’\mathrm{ct}\right)}{R’\mathrm{ct}}\times 100$$

where Rct and R’ct denote the charge transfer resistance values with and without different concentrations of the inhibitor, respectively. 

## 3. Results and Discussion

### 3.1. Surface Analysis

**Figure 1:**
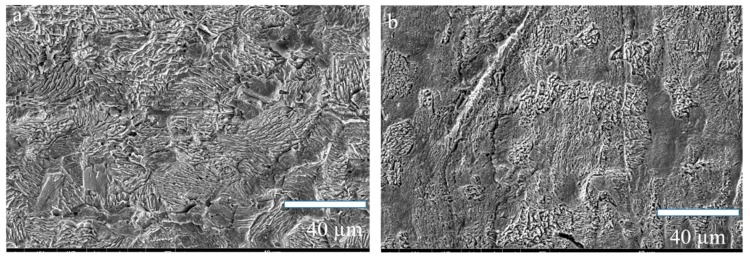
SEM micrographs of the Fe after submersion in 1.0 mol/L (**a**) HCl and 75 mg/L lignin-based quaternary ammonium material (lignin-DMC) + 1.0 mol/L HCl (**b**).

The synthesis and characterization of lignin-DMC was presented in supporting information. The synthesis process was shown in Figures S1 and S2, briefly, 2.0 g of lignin were dissolved in water in a 250 mL three-neck glass flask. DMC (lignin and DMC chloride molar ratio of 1:1.6) was added to the solution and the pH of the medium was adjusted to 4–5. K_2_S_2_O_8_ (0.03 g) was then dissolved in 5 mL of deionized water and added drop wisely to the suspension. Then, the polymerization was conducted at 80 °C for 3 h, after completion, the polymerization’s solution was further purified by precipitation with 200 mL of ethanol and centrifuging. The lignin-DMC polymer contains the characteristic absorption peak of lignin and DMC, confirming that the lignin-DMC was successfully synthesized (Figure S3). In Tables S1 and S2, the molecular weight of the lignin-DMC polymer was significantly higher than that of kraft lignin, besides, the nitrogen contents of the lignin-DMC polymer were higher than that of kraft lignin, which were in accordance with the FT-IR analysis (Figure S3). Figure 1 presents the SEM images of the samples with and without corrosion inhibitor by immersion in 1.0 mol/L HCl medium for 2 h. Samples without a corrosion inhibitor showed a rough and uneven state (Figure 1a), indicating that the iron was seriously corroded. However, the surfaces of the materials immersed in the HCl medium containing lignin-DMC were relatively flat (Figure 1b), showing that the corrosion degree was greatly reduced and that the lignin-DMC displayed a significant inhibitory effect in HCl. In addition, micrographs of steel without HCl and Fe after submersion in the 100 mg/L lignin-DMC + 1.0 mol/L HCl was shown in Figure S4.

**Figure 2:**
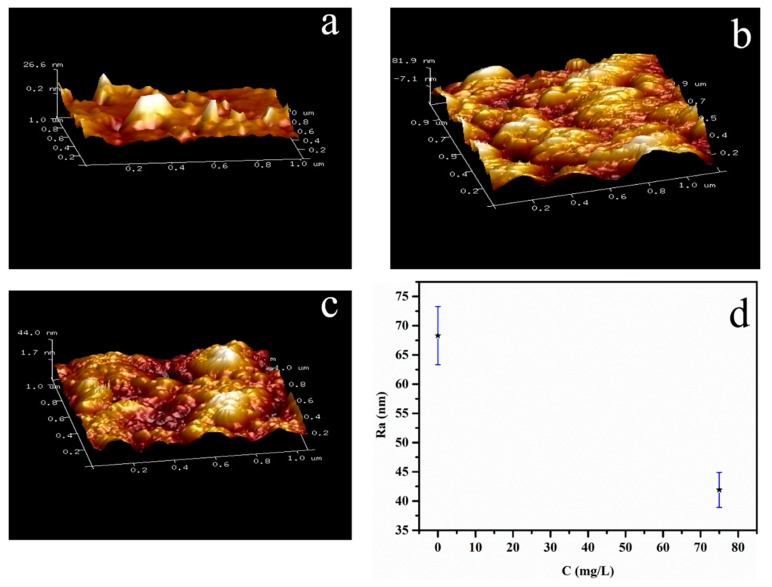
Images of the Fe surface (**a**), Fe after submersion in 1.0 mol/L (**b**) HCl, 75 mg/L lignin-DMC + 1.0 mol/L HCl (**c**) and (**d**) the average roughness (Ra) of the Fe surface in (**b**,**c**).

For research, the roughness of the material surface clearly, and the AFM tests of the samples without and with the inhibitor by immersion in 1.0 mol/L HCl medium for 0.5 h were shown in Figure 2. The sample without the inhibitor, the surface of Fe shows rough and inhomogeneity. The average roughness (Ra) for the samples without a corrosion inhibitor is 81.9 nm. However, lignin-DMC displayed that the surface was smooth. This roughness has been reduced to 44 nm with the inhibitor. This result shows that the inhibitor plays a role in anticorrosion behavior.

### 3.2. Weight Loss Experiment

The weight loss measurement, which is an easy and convenient method, was used first for initially testing the anti-corrosion performance of the inhibitor. Table 1 shows the CR of iron in a 1.0 mol/L HCl at 25 °C and the inhibition rate (*η_IE_*_)_ of different concentrations of the lignin-DMC inhibitor (c) (weight loss test). For the blank solution, absent of the corrosion inhibitor, a large amount of H_2_ was produced when the iron was exposed to the HCl, and the corrosion rate was as high as 2.65 mg/ (cm^2^·h). However, the corrosion rate decreased immediately and showed a tendency of first decreasing and then increasing after adding the lignin-DMC. When the concentration was 75 mg/L, the corrosion rate was the lowest and decreased to 0.33 mg/(cm^2^·h). On the other hand, the corrosion inhibition rate expressed the opposite trend. At 75 mg/L, the corrosion inhibition rate was the highest at 87.65% and at 50 mg/L was the lowest at 70.39%. These results exhibited that the lignin-DMC can be an efficient inhibitor in an HCl medium.

### 3.3. Electrochemical Measurements

#### 3.3.1. Open Circuit Potential

**Figure 3:**
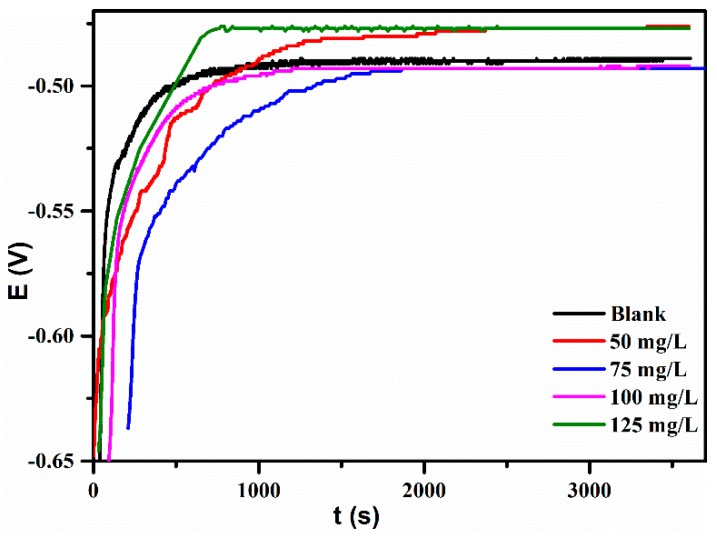
OCP-time curves for iron with and without lignin-DMC at 25 °C.

Figure 3 shows the *OCP* immersion time (t) at 25 °C for iron in HCl with and without various conventions of lignin-DMC. It can be seen that the open circuit potential gradually increases over time and gradually stabilizes. A blank HCl solution without a corrosion inhibitor began to reach a relatively stable state at 1000 s. However, the HCl solution containing 75 mg /L lignin-DMC reached a final equilibrium state at after 2000 s. The potential polarization curves and electrochemical impedance spectroscopy (EIS) test achieved a stable state after an immersion time of 1 h. 

#### 3.3.2. Polarization Studies

**Figure 4:**
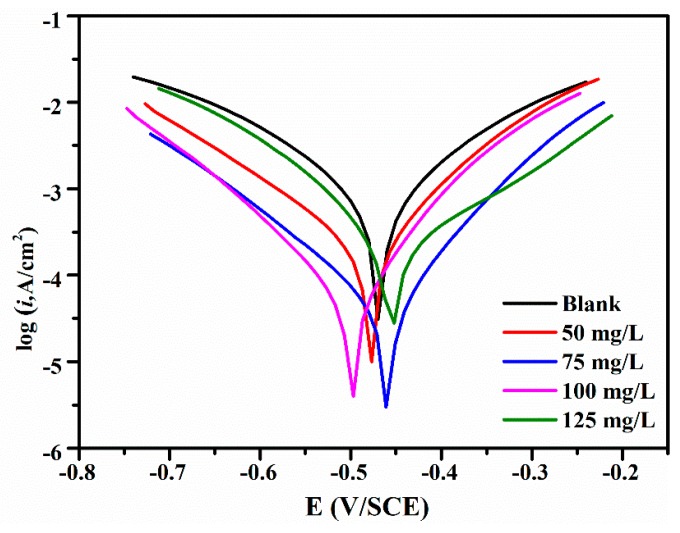
Polarization curves in a 1.0 mol/L HCl solution containing various inhibitor concentrations at 25 °C.

The IVIUM electrochemical workstation was used to determine the polarization curves at several different concentration of lignin-DMC. In the Tafel curves, the vertical axis is the logarithm of the corrosion current density and the horizontal axis is the corrosion potential. The results are shown in Figure 4. The polarization curves generated by the electrochemical parameters values, including the corrosion current density (Icorr), corrosion potential (Ecorr), the anodic Tafel slope (ba), the cathodic Tafel slope (bc), and the inhibit efficiency in 1.0 mol/L of HCl with and without various concentrations of lignin-DMC are presented in Table 2. The data in Table 2 indicates that at a concentration of 75 mg/L lignin-DMC the corrosion current density was at its relative lowest, which is a satisfactory agreement with the weight loss measurement. The addition of the inhibitor molecules at different concentrations did not cause a significant change in the cathode and anode curves, and the polarization curves at each concentration were substantially parallel to the Tafel curve of the blank solution. It indicates that the addition of the compound does not change the metal anode dissolution and the cathode hydrogen evolution reaction. The corrosion inhibition mechanism only inhibits the active point of the reaction by forming a protective film on the surface of the carbon steel. With various concentrations of the inhibitor, the Tafel slope of the anode of the polarization curve increased from 94 mV to 154 mV, and the Tafel slope of the cathode increased from 114 mV to 185 mV, indicating that a certain inhibitory effect of the corrosion inhibitor occurred on the iron of the anode and cathode reactions, but the cathode Tafel slope increased more rapidly than the anode Tafel slope did, further proving that the lignin-DMC belongs to an inhibition mixed-type inhibitor. The displacement in the Ecorr value is less than 85 mV, which is also evidence for the lignin-DMC acting as a mixed type inhibitor [32]. 

#### 3.3.3. EIS Studies

**Figure 5:**
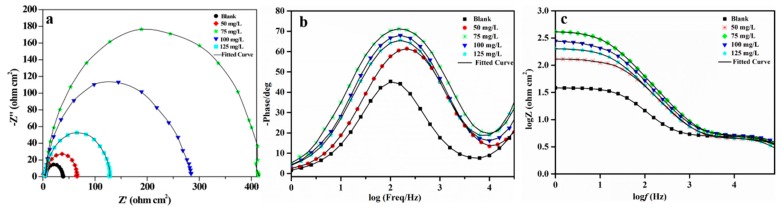
(**a**) Nyquist plots, (**b**) bode plots and phase angle plots (**c**) of steel in an HCl solution containing different inhibitor concentrations.

Electrochemical impedance spectroscopic (EIS) method allows for the assessment of the performance of adsorbed inhibitor film against the corrosion kinetics [21]. Electrochemical impedance spectroscopy can quickly evaluate the corrosion resistance and obtain the electrochemical information of the metal under the film that was formed by lignin-DMC adsorption on the surface of iron. High-frequency is corresponding to the layer information and the high-frequency capacitive reactance further responses to the dielectric properties and shielding performance of the film layer. A low-frequency end can give information on the metal/solution interface reaction, and the size of the low-frequency capacitance represents the metal charge transfer resistance during corrosion. The Nyquist and Bode diagrams in the HCl and solutions contained in the lignin-DMC are shown in Figure 5. The impedance spectrum is mainly composed of a single high-frequency end-capacity arc, which is characterized by only one time constant. The single capacitive reactance arc change indicates that the electrode surface corrosion process is mainly controlled by the charge transfer step. The semicircle size increased after adding the inhibitors, which was due to the charge transfer [45]. With an increase in the lignin-DMC concentration, the size increased [46,47]. Increase in Rct values with composite concentration within the acid electrolyte is consistent with the formation of polymer film on the metal surface due to charge transfer action. At a concentration of 75 mg/L, the semicircles size showed their largest value. 

**Figure 6:**
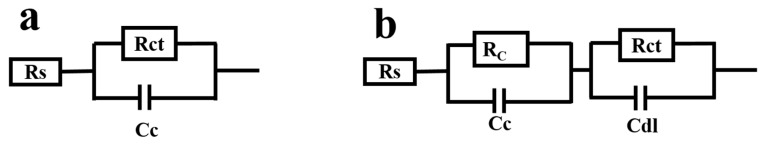
The electrical equivalent circuit of the capacitive loop for electrochemical impedance spectroscopy (EIS) without inhibitor (**a**) and the electrical equivalent circuit in different concentration of inhibitors at 25 °C (**b**).

The interface layer between the electrode and the solution, also known as the electric double layer, is generally represented by an equivalent capacitance. The equivalent circuit shown in Figure 6 was used to simulate the impedance data with the values shown in Table 3, where *R_s_* acts as the charge solution resistance. The blank solution and 50 mg/L fitted (1), and the rest fitted (2). R_c_ acts as the film resistance. In addition, R_ct_ represents the charge transfer resistance, C_c_ is the film capacitance of the double layer, and Cdl are the electric double layer capacitors. At a high frequency the membrane resistance became larger and the membrane capacitance decreased, indicating that after the addition of the lignin-DMC in the HCl solution the lignin-DMC molecules removed the water molecules, which were originally adsorbed onto the iron-based material, thereby creating a shielding effectiveness and arrived at a protective effectiveness. As the radius of the capacitive arc of the low frequency increased, the charge transfer value R_ct_ became larger, and the charge transfer capacitance decreased. The increase in R_ct_ was due to the considerable surface coverage by the inhibitor molecules on the metal surface through bonding. The decrease in Cdl can be explained by a decrease in the local dielectric constant and/or an increase in the thickness of the electrical double layer, which indicates the adsorption of the inhibitor [48]. Hence, the lignin-DMC had an excellent inhibitory effectiveness on the iron corrosion in acidic media. 

### 3.4. Effect of Temperature

**Figure 7:**
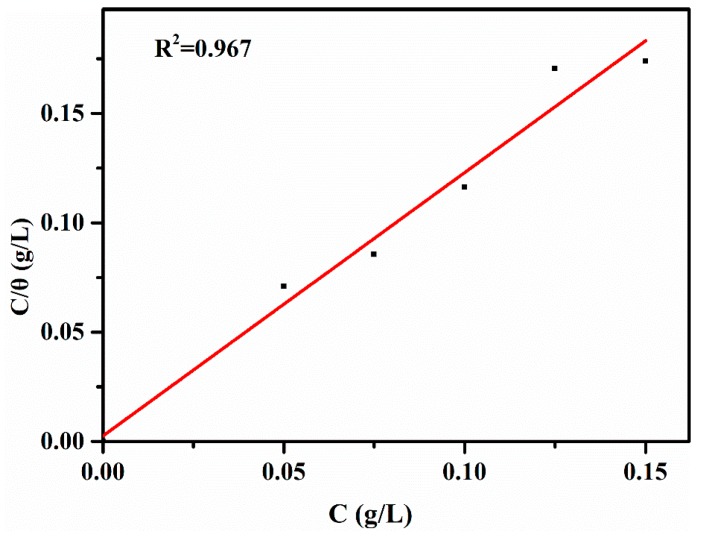
Langmuir adsorption model of iron in 1 mol/L HCl solution at room temperature.

Adsorption thermodynamics and adsorption kinetics are effective research methods for determining the corrosion inhibitor adsorption behavior. To attain a better performance of the lignin-DMC of adsorption and activation processes, the effect of the temperature was studied. By studying the adsorption isotherm model and the corresponding formula, the corresponding adsorption parameters, such as the adsorption equilibrium constant and adsorption free energy, can be calculated to investigate the adsorption mechanism of this corrosion inhibitor. The values of the surface coverage (θ) are defined and calculated as IE/100 from the weight loss measurements using the following equation [41]: Several isothermal adsorption methods were matched by the experimental results, following the Langmuir adsorption isotherm model. The adsorption isotherm is as follows and the plot is shown in Figure 7.

(5) $$\frac{C}{\theta }\;=\;\frac{1}{K}\;+\;C$$

θ represents the surface coverage. K is the equilibrium constant and C is the concentration of the inhibitor, mg/ L.

The inhibitor molecules gradually adsorb onto the metal surface by changing the adsorbed water molecules. The free energy of the adsorption ΔG was calculated based on Equation (6):

(6) $$K\;=\;\frac{1}{55.5}\;\exp\;(\frac{-∆G}{\mathrm{RT}})$$

where ΔG is the free energy of adsorption, K is the adsorption–desorption equilibrium constant, R is the universal gas constant, and T is absolute temperature in Kelvin, K.

The Langmuir isotherm adsorption model of lignin-DMC in 1 mol/L HCl at 25 °C is shown in Figure 7. The values of ΔG were −27.51 kJ/mol, −28.42 kJ/mol and −29.33 kJ/ mol at temperatures of 25, 35 and 45 °C, respectively, with the inhibitor at 75 mg/L. Negative values indicate that the adsorption was spontaneous. If the absolute value of G is approximately 20 kJ/mol, the adsorption mode then belongs to the physical adsorption. Additionally, the adsorption mode follows chemical adsorption if the absolute value of ΔG is approximately 40 KJ/mol. Physical adsorption and chemical adsorption (mixed adsorption) would occur if the values are between 20 to 40 kJ/mol [6]. Hence, the adsorption mode of lignin-DMC was physical adsorption and chemical adsorption.

The relationship between the temperature and corrosion rate is expressed by the following equation and its alternative formulation, called the transition state equation, was applied to determine the activation entropy (ΔS) and activation enthalpy (ΔH)

(7) $$\ln(\mathrm{CR})=A\;\exp\;\frac{-E_{a}}{\mathrm{RT}}$$

The transition state equation is as follows:

(8) $$\mathrm{CR}=\frac{\mathrm{RT}}{\mathrm{Nh}}\;\exp\frac{\Delta S}{R}\;\exp\;\frac{-\Delta H}{\mathrm{RT}}$$

where CR is the corrosion rate, A is the Arrhenius pre-exponential constant, R is the universal gas constant (8.314 J/mol/K), and h is Plank’s constant (6.63 ×10^34^ J·s) [48].

**Figure 8:**
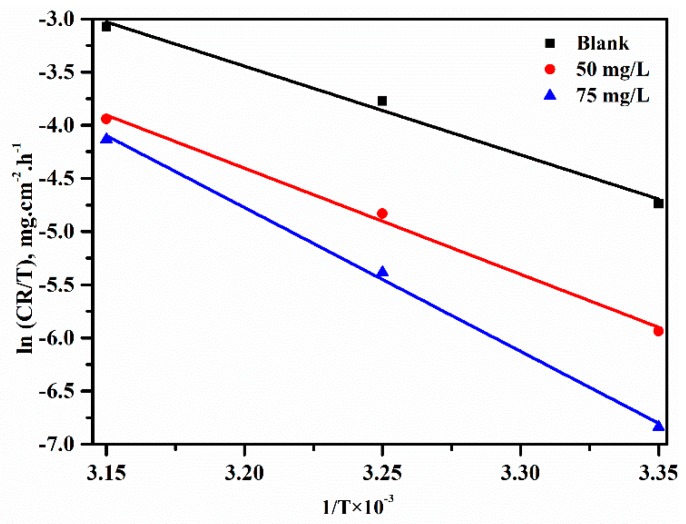
Arrhenius plots of the corrosion rate (CR/T) in an HCl solution in the absence and presence of Lignin-DMC.

The plot of CR/T vs 1/T is expressed in Figure 8, and the values of the activation enthalpy (ΔH) and the activation entropy (ΔS) were obtained by the following equation:

(9) $$\Delta H\;=\;-\mathrm{slope}\;\times \;R\;\mathrm{and}\;\Delta S\;=\;(\mathrm{intercept}\;-\;\ln[\frac{R}{\mathrm{Nh}}])\cdot R$$

The values of ΔH were 69.29 KJ/mol, 82.93 KJ/mol and 112.4 KJ/mol at different concentrations, which indicated it was an endothermic process. The values of ΔS were 146.5 KJ/mol 182.1 KJ/mol and 273.5 KJ/mol, respectively. The value increased (more positive) in the presence of the lignin-DMC compared to the blank solution, which illustrated that the system passes from a less orderly to a more random arrangement [49]. The results may be explained by the adsorption of organic inhibitor molecules and considered to be a quasi-substitution process between the organic inhibitor molecules in the aqueous phase and the water molecules on the surface of low carbon steel. The adsorption process of the inhibitors was the displacement reaction of the adsorption water molecules removed from the metal surface [50].

Org(sol) + *n*H_2_O (ads) → Org(ads)+ *n*H_2_O (10)

Org(sol) and Org(ads) are organic molecules that are dissolved in the solution and adsorbed onto the iron surfaces. H_2_O (ads) are the water molecules on the metal’s surface, where *n* is the factor that indicates the substitution of the water molecules by the inhibitor units [39].

### 3.5. Mechanism of the Corrosion Inhibitor

**Figure 9:**
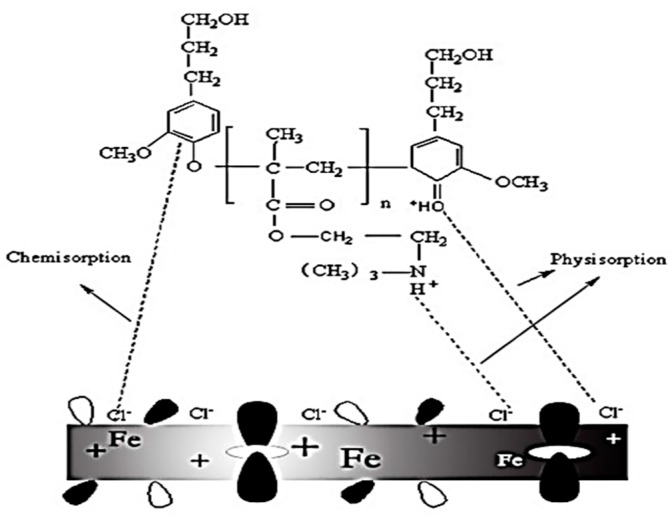
Schematic representation of lignin-DMC with the metal surface in 1 mol/L HCl.

Lignin-DMC is composed of polar groups consisting mainly of N and O atoms with higher electronegativity and nonpolar groups consisting of C and H non-polar atoms, which can be adsorbed onto the steel surface to change electric double layer structure of the metal and improve the activation energy of the metal ionization process. Nonpolar groups move away from the metal surface to form a layer of hydrophobic film, impeding the transfer of charges and substances and thus greatly reducing the metal corrosion rate. The mechanism of the corrosion inhibitor is shown in Figure 9. In HCl solutions, the unshared electron pair at the central atom of the organic corrosion inhibitor formed an onium ion with a hydrogen ion in HCl solutions. Under the effect of the electrostatic attraction, the onium iron was adsorbed onto the cathode region of the metal surface (Cl^−^), making the metal surface seem positive. The onium irons then began to compete with H^+^ [16], so the H^+^ ions in the acid solution have difficultly moving close to the metal, which greatly slows down the corrosion rate. Another adsorption mode was chemisorption. In this adsorption, the central atoms of the polar group of the organic corrosion inhibitor molecules, N and O, have unshared electron pairs. When the metal has an empty orbit, the lone pair of electrons at the central atoms of the polar groups may combine with the empty orbitals to form coordination bonds, so the lignin-DMC are adsorbed onto the steel surface to slow down the corrosion rate.

## 4. Conclusions

Cationic lignin-based polymer (Lignin-DMC) was synthesized by copolymizing DMC with kraft lignin. The prepared lignin-DMC was an effective corrosion inhibitor in the 1 mol/L HCl solution. The corrosion inhibition effect was best at 75 mg/L at room temperature, reaching a state at 87.65%. Meanwhile, the results revealed that the proper dosage may be used as an inhibitor. However, a superfluous dosage had a negative effect on the anticorrosion performance. Additionally, the corrosion inhibitor-lignin-DMC was a mixed corrosion inhibitor. The adsorption mode of lignin-DMC was the Langmuir adsorption isotherm. The mechanism of adsorption was found to be a physical and chemical model in nature. The adsorption model indicated that the corrosion inhibitor was an adsorption type corrosion inhibitor

## Figures and Tables

**Table 1:** Corrosion parameters and IE (Inhibition Efficiency) obtained from weight loss of iron-based materials in a 1.0 mol/L HCl at different concentrations of lignin-DMC at 25 °C.

Concentration (ppm)	CR (mg /(cm^2^·h))	IE (%)
0	2.65	0.00
50	0.80	69.81%
75	0.33	87.54%
100	0.37	86.04%
125	0.71	73.21%

**Table 2:** Different electrochemical parameters of iron sheet in 1.0 mol/L HCl solutions at various lignin-DMC concentrations.

C (mg/L)	Ecorr (mV/SCE)	icorr (uA/cm^2^)	ba (mV)	bc (mV)	IE (%)
**0**	−461.80	891	154	185	0.00
**50**	−427.60	246	164	148	72.39%
**75**	−460.30	45	94	114	94.95%
**100**	−508.40	78	104	125	91.25%
**125**	−488.30	231	122	147	74.07%

**Table 3:** Fitted EIS results of iron corroding in 1 mol/L HCl solutions at different lignin-DMC.

Concentration(mg/L)	R_s_(ohm)	C_c_(uF)	R_c_(ohm)	R_ct_(ohm)	Cdl(uF)	IE(%)
0	5.729	–	–	29.60	254.60	–
50	4.701	–	–	59.74	240.60	50.45%
75	5.508	91.19	178.90	227.00	34.09	86.96%
100	4.760	180.70	96.97	101.00	45.91	70.69%
125	4.780	231.90	50.32	74.24	42.70	60.12%

## Supplementary Materials

The following are available online at https://www.mdpi.com/1996-1944/12/11/1776/s1, Figure S1. Schematic diagram of a lignin-DMC synthesis device, Figure S2. (a) The reaction mechanism and (b) The sketch of the lignin grafting reaction, Figure S3. FT-IR spectra of kraft lignin and lignin-DMC polymer, Figure S4. SEM micrographs of the Fe surface without 1.0 mol/L HCl (a), Fe surface after submersion in 1.0 mol/L HCl (b), 1.0 mol/L HCl with the addition of 75 mg/L lignin-DMC (c), and (d) Fe immersed in 1.0 mol/L HCl + 100 mg/L Lignin-DMC solutions, Table S1. Molecular weight and charge density of lignin, and lignin-DMC polymer, Table S2. Elemental analysis of lignin and lignin-DMC polymer.
