# Supplementary material for: Construction of a Novel Lignin-Based Quaternary Ammonium Material with Excellent Corrosion Resistant Behavior and Its Application for Corrosion Protection
Source: Materials (Basel). 2019 May 31;12(11):1776. doi: 10.3390/ma12111776 (PMC6600727; doi:10.3390/ma12111776)
Supplement: Supplementary file 1 [file materials-12-01776-s001.pdf]

materials

Supporting Information

Construction of a Novel Lignin Based Quaternary Ammonium Material With Excellent Corrosion Resistant Behavior and Its Application for Corrosion Protection

Chao Gao, Shoujuan Wang, Xinyu Dong, Keyin Liu, Xin Zhao * and Fangong Kong *

State Key Laboratory of Biobased Material and Green Papermaking, Key Laboratory of Pulp & Paper Science and Technology of Shandong Province/Ministry of Education, Shandong Academy of Sciences, Qilu University of Technology, Jinan 250353, China; 15864029637@163.com (C.G.); nancy5921@163.com (S.W); d18954536225@163.com (X.D.); keyinliu@163.com (K.L.)

* Correspondence: zhaoxin_zixi@126.com (X.Z.); kfgwsj1566@163.com (F.K.); Tel.: +86-531-896-319-88 (F.K.)

1. Materials and Experimental Method

1.1. Synthesis and Purification of Lignin-DMC

In these experiments, 2.0 g of lignin were dissolved in water in a 250 mL three-neck glass flask. The flask was immersed into a water bath and the suspension was deoxygenated by purging with nitrogen gas for 30 min. DMC (lignin and DMC chloride molar ratio of 1:1.6) was added to the solution and the pH of the medium was adjusted to 4–5. $K_2S_2O_8$ (0.03 g) was then dissolved in 5 mL of deionized water and added drop wisely to the suspension. The total volume of the reaction medium was 35 mL, which contained a lignin concentration of 0.3 mol/L (all the lignin molar concentrations reported in this paper are calculated based on the lignin C_9 unit using a molecular weight of 185 g/mol). The polymerization was conducted at 80 °C for 3 h. A continuous supply of nitrogen was maintained throughout the reaction. After completion of the reaction, the solution was cooled to room temperature and the solution was mixed with 200 mL of ethanol in order to precipitate the polymer. The suspension was then centrifuged at 3500 rpm for 10 min (Sorvall ST 16 Laboratory Centrifuge, Thermo Fisher, in order to separate the lignin-graft-DMC polymer from the suspension. During the experiments, a homopolymer (PDMC) was produced. Upon completion, the pH of the solution was adjusted to 7.0 using 0.1 mol/L NaOH, and then dialyzed for 48 h. The water for the dialysis was changed every 4 h in the first 24 h and every 6 h for another 24 h. The solution from the dialysis was dried at 105 °C for 24 h and considered as lignin-DMC in this paper.

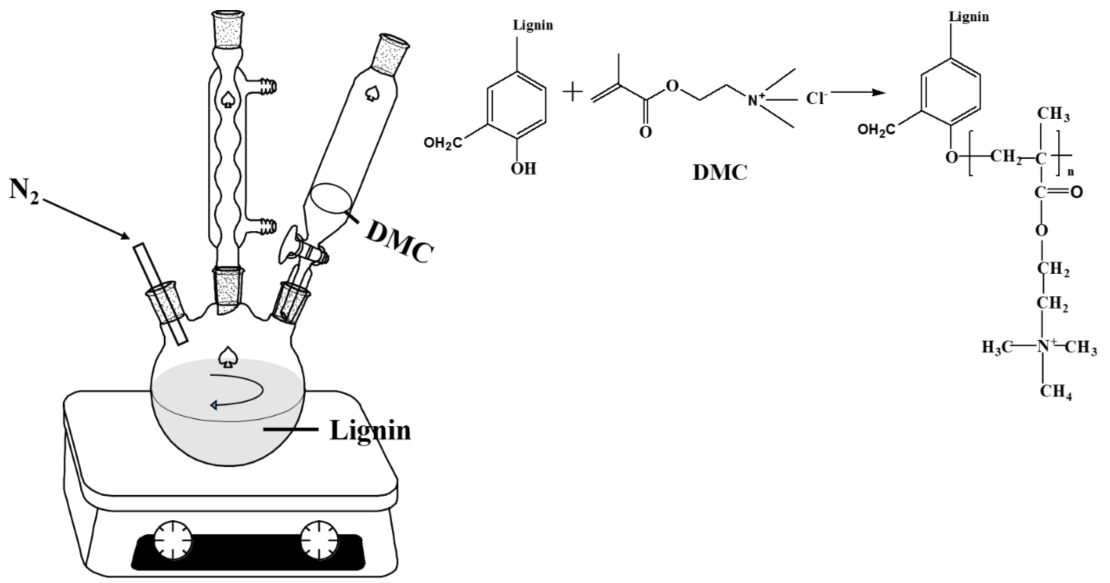

Figure S1. Schematic diagram of a lignin-DMC synthesis device.

1.2. Characterization

1.2.1. Charge Density Analysis

Approximately 0.05 g of the lignin-DMC polymer was dissolved in 50 g of water, and the solution was immersed in a water bath shaker (Innova 3100, Brunswick Scientific, Edison, NJ, USA) and shaken (100 rpm) at 30 °C for 1 h. The suspension was then centrifuged at 1000 rpm for 5 min. The supernatant was collected and used for the charge density analysis. The charge density of lignin-DMC polymer was measured using a Particle Charge Detector, Mütek PCD 04 titrator (Arzbergerstrae, Herrsching, Germany) with a PVSK solution of 0.005 mol/L. The reported data in this paper is the average of three repetitions. Furthermore, the concentration of the lignin-DMC polymer in the supernatant was determined by drying the supernatant at 105 °C overnight.

1.2.2. Elemental Analysis

Kraft lignin used in the study had a very low nitrogen content. The nitrogen content of the lignin-DMC polymer originated from the DMC attached to lignin. The elemental analysis was performed with an Elementar Vario EL (Germany) elemental analyzer.

1.2.3. Molecular Weight Analysis

For the kraft lignin analysis, approximately 10 mg of air-dried lignin was acetylated using 2.5 mL of acetyl bromide/acetic acid 8/92 (V/V) at 50 °C for 2 h. Then the solvent was removed using a freeze dryer. The acetylated lignin was dissolved in 10 mL of tetrahydrofuran (THF) and filtered with a PTFE 13 mm diameter filter with a pore size of 0.2 μm. The filtered samples were used in the molecular weight analysis using a high-performance liquid chromatography system, Agilent Model 1200 with a UV diode-array detector, multiangle laser light scattering detector and an RI detector. Waters Styragel HR4 (WAT044225), HR4E (WAT044240) and HR1 (WAT 044234) columns and precolumns (P/N WAT054405) were used and the flow rate of THF was set at 1.0 mL/min. The column temperature was set at 25 °C and polystyrene polymers were used as standards. For the lignin-DMC polymer analysis, 2 mg of the air dried polymer sample were dissolved in 10 mL of a 5% acetic acid solution by stirring at 300 rpm for 6 h and then filtered with a nylon 0.2 μm filter (13 mm diameter). The filtered solutions were used for molecular weight analysis using a gel permeation chromatography system, Malvern GPC max VE2001 Module and Viscotek TDA305 with multidetectors (UV, RI, viscometer, low angle and right angle laser detectors). The columns of

PAC103 and PAC101 were used with a 0.50 mL/min flow rate using a 5% acetic acid solution. The column temperature was set at 35 °C. Pullulan with a molecular weight of 47, 300 g/mol was used as the standard.

1.2.4. Fourier Transform Infrared (FT-IR)

The FT-IR spectra of lignin and lignin-DMC were recorded using a VERTEX 70 FT-IR spectrophotometer. Each spectrum was recorded in transmittance mode with 32 scans in the frequency range of 400 and 4000 cm^{-1} with a 4 cm^{-1} resolution.

2. Results and discussion

2.1. Reaction Mechanism

The mechanism of the lignin grafting reaction is shown in Figure S1. Three main reactions, including the initiation reaction, chain propagation reaction and termination reaction occurred in this system. In this reaction, the sulfate radicals can initially be formed by heat decomposition, which can take unstable hydrogen from the phenolic hydroxyl groups of lignin to generate phenoxy radicals. These phenoxy radicals can also form resonance radicals. The phenoxy radicals and its resonance radicals are formed on the lignin backbone (free radical reaction sites); and then react with the monomer DMC or propagated monomer to form a lignin polymer, as shown in reaction of FigureS1. The poly DMC chain segments, which contain quaternary ammonium groups existing on lignin-DMC polymer, would offer cationic charges, water solubility and a higher molecular weight to the lignin.

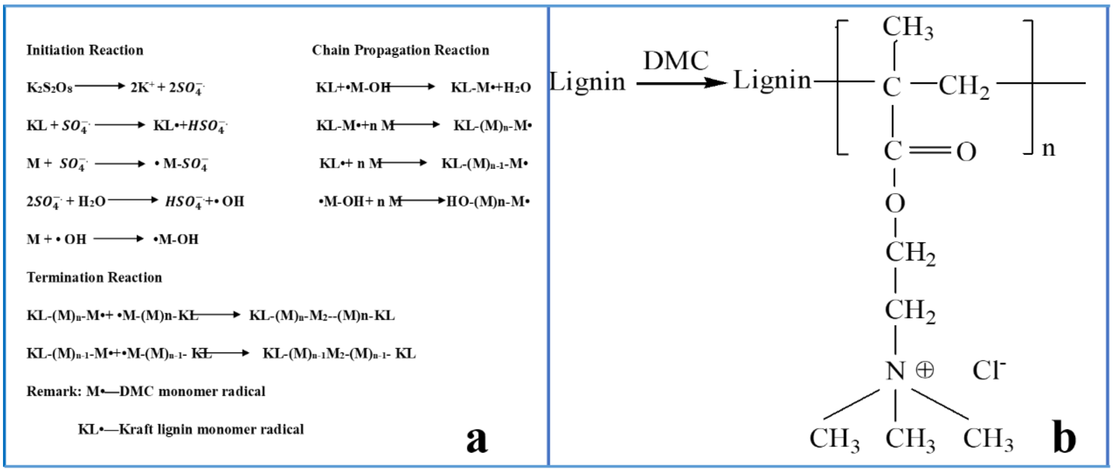

Figure S2. (**a**) The reaction mechanism and (**b**) The sketch of the lignin grafting reaction.

2.2. Charge Density and Molecular Weights Analysis

The molecular weight and charge density of the kraft lignin and lignin-DMC polymer are listed in Table S1. The molecular weight of the lignin-DMC polymer was 1.53×10^6 g/mol, which was significantly higher than that of kraft lignin (26,100 g/mol), demonstrating the successful polymerization of DMC and lignin.

Table S1. Molecular weight and charge density of lignin, and lignin-DMC polymer.

Sample	lignin	lignin-DMC polymer
M_n, g/mol	17,300	1.14×10^6
M_w, g/mol	26,100	1.53×10^6
M_w/M_n	1. 510	2.740
Charge density, meq/g	–	2.93

2.3. FT-IR Analysis

Figure S2 presents the spectra of lignin and the lignin-DMC polymer. A broad characteristic absorption peak appeared at the wavenumber of 3446.3 cm^{-1}, which was caused by the stretching vibration of the hydroxyl group (-OH). The band of two samples at 2894.6 cm^{-1} corresponds to the C-H group. In summary, the basic characteristics of lignin have not been destroyed after the copolymerization of lignin with DMC. However, the infrared spectra of the lignin-DMC polymer was also significantly different from that of the original lignin. A peak at 1716.8 cm^{-1} occurred in the infrared spectrum of the lignin-DMC polymer, which was the absorption peak of the acyloxy group. Additionally, the characteristic absorption peak of the quaternary ammonium group $-CH_2-N^+(CH_3)_3$ appeared at 956.7 cm^{-1}. The results exhibited that the lignin-DMC polymer contains not only the absorption peak of lignin but also the characteristic absorption peak of DMC, confirming that the Lignin-DMC was successfully synthesized by the grafting method.

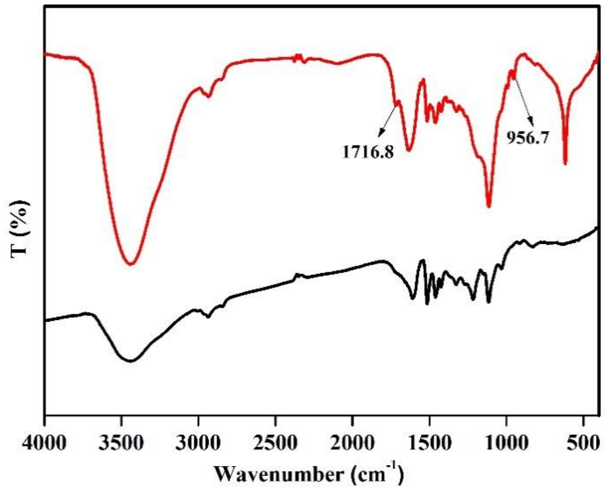

Figure S3. FT-IR spectra of kraft lignin and lignin-DMC polymer.

2.4. Elemental Study

The elemental analysis of lignin and lignin-DMC are listed in Table S2. It was clear that the carbon and oxygen contents of the lignin-DMC polymer were lower than that of kraft lignin, which is attributed to the lower carbon and oxygen contents in the DMC segment of the lignin-DMC polymer. The nitrogen content of the polymer was 4.32%, but that of kraft lignin was 0.01. These changes confirmed the polymerization of lignin and DMC. The C_9 unit formulas of lignin and lignin-DMC were also determined based on the elemental analysis (Table. S2).

Table S2. Elemental analysis of lignin and lignin-DMC polymer.

Sample	N, wt.%	C, wt.%	H, wt.%	O, wt. %	Formula
Lignin	0.01	60.45	5.97	31.40	$C_9H_{10.67}O_{3.51}N_0$
Lignin-DMC	4.32	55.14	7.71	21.18	$C_9H_{15.1}O_{2.59}N_{0.6}$

2.5. SEM Analysis

The micrographs of Steel without HCl (a) and Fe after submersion in the 100 mg/L lignin-DMC + 1.0 mol/L HCl (b) was shown in Figure S4. And at the 100 mg/L, the surface of steel exhibit more severe corrosion than 75 mg/L.

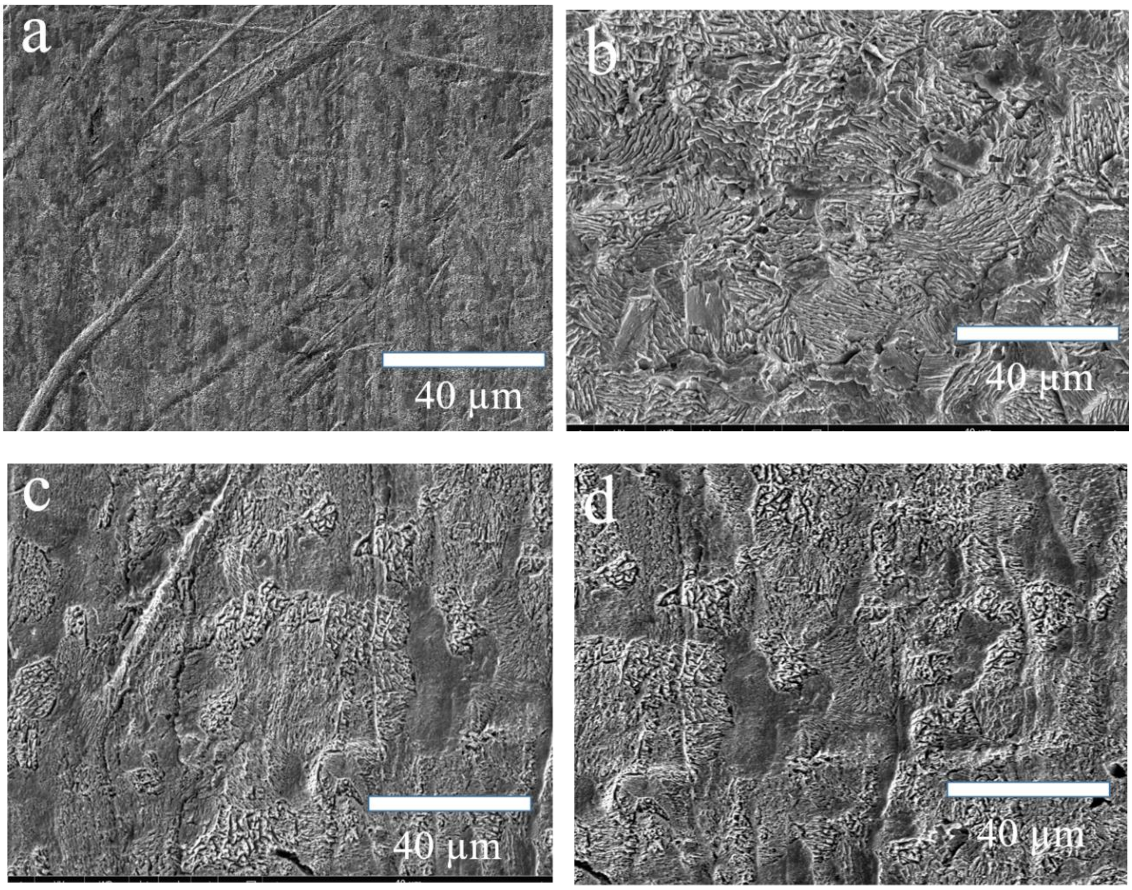

Figure S4. SEM micrographs of the Fe surface without 1.0 mol/L HCl (**a**), Fe surface after submersion in 1.0 mol/L HCl (**b**), 1.0 mol/L HCl with the addition of 75 mg/L lignin-DMC (**c**), and (**d**) Fe immersed in 1.0 mol/L HCl + 100 mg/L Lignin-DMC solutions.

© 2019 by the authors. Submitted for possible open access publication under the terms and conditions of the Creative Commons Attribution (CC BY) license (http://creativecommons.org/licenses/by/4.0/).
